# Needs of caregivers in heart failure management: A qualitative study

**DOI:** 10.1177/1742395315574765

**Published:** 2015-12

**Authors:** Jennifer Wingham, Julia Frost, Nicky Britten, Kate Jolly, Colin Greaves, Charles Abraham, Hayes Dalal

**Affiliations:** 1 BIU, Knowledge Spa, Royal Cornwall Hospital, Truro, UK; 2 University of Exeter Medical School, Primary Care Research Group, Exeter, UK; 3 Institute of Health Research, University of Exeter Medical School, Exeter, UK; 4 University of Birmingham, Birmingham, UK; 5 Psychology Applied to Health Group, University of Exeter Medical School, Exeter, UK

**Keywords:** Heart failure, caregiver, needs, qualitative, Rehabilitation Enablement in Chronic Heart Failure

## Abstract

**Objectives:**

To identify the needs of caregivers supporting a person with heart failure and to inform the development of a caregiver resource to be used as part of a home-based self-management programme.

**Methods:**

A qualitative study informed by thematic analysis involving 26 caregivers in individual interviews or a focus group.

**Results:**

Three distinct aspects of caregiver support in heart failure management were identified. Firstly, caregivers identified needs about supporting management of heart failure including: coping with the variability of heart failure symptoms, what to do in an emergency, understanding and managing medicines, providing emotional support, promoting exercise and physical activity, providing personal care, living with a cardiac device and supporting depression management. Secondly, as they make the transition to becoming a caregiver, they need to develop skills to undertake difficult discussions about the role; communicate with health professionals; manage their own mental health, well-being and sleep; and manage home and work. Thirdly, caregivers require skills to engage social support, and voluntary and formal services while recognising that the long-term future is uncertain.

**Discussion:**

The identification of the needs of caregiver has been used to inform the development of a home-based heart failure intervention facilitated by a trained health care practitioner.

## Introduction

Heart failure (HF) significantly affects the quality of life of patients living with the condition and is a challenge for primary and secondary care.^1,2^ It also has an impact on the physical and mental health of caregivers (family or friends) as they support self-management strategies or provide personal care.^1,3–5^ Specific caregiving activities in HF may include general day-to-day monitoring of well-being and changes in health status, supporting adherence to dietary restrictions, planning and pacing of daily activities and a complex medication regime that frequently requires modification in the context of daily life, taking emergency measures such as knowing when to call a doctor, inclusion in social activities, ensuring safety, emotional support and end-of-life care.^4,6^

The new UK Care Act 2014^7^ sets out the statutory rights of caregivers to be assessed *and* supported and has implications for both health and social care. In addition, the 2010 National Institute of Health and Care Excellence clinical guidelines 108 for HF also recommended that family members or caregivers are included in discussions about care.^8^ Despite this recommendation, lay caregivers often have poor knowledge or receive little advice on developing their abilities to support self-care.^9,10^

In a recent trial from Sweden,^11^ 155 HF patients and their caregivers were randomised to either usual care or intervention (involving three nurse sessions for patients and caregivers and education via leaflets and a Compact Disc) in addition to usual care. There were no differences at 12-month follow-up in carers’ health behaviour, confidence or burden when compared with usual care. In contrast, Piamjariyakul et al.^12^ found in a pilot study in the United States that a telephone coaching programme for HF caregivers with supporting written materials was rated to be helpful by the caregiver participants. There have been calls for the development of more effective HF interventions that include caregivers in order to improve quality of care, reduce costs and improve patient quality of life,^5,13^ and it has been suggested that caregivers be included in the early stages of rehabilitation.^1,5^ It has also been argued that the development of effective interventions of caregivers of people with HF needs detailed qualitative research to better understand the complexity of cared-for and caregiver relationships and the impact on self-management.^14^

The study reported in this paper helped to inform the development of an intervention for caregivers as part of the Rehabilitation Enablement in Chronic Heart Failure (REACH-HF) research programme.^15^ This is a home-based intervention designed to improve self-management skills and quality of life in people with HF. It includes a HF manual and a family and friends resource comprising work books facilitated by a trained health care practitioner tailored to individuals’ specific needs. Prior to this research, members of the REACH-HF team conducted a qualitative synthesis of patient perspectives on managing HF that informed the development of the HF manual and the design of the research presented here.^16^ The aim of this study was to undertake a qualitative assessment of the needs of HF caregivers to inform an evidence-based self-help intervention (‘REACH-HF manual’) aimed at HF patients and caregivers.

## Methods

### Design

Semi-structured face-to-face interviews were thematically analysed^17^ and a subsequent focus group with caregivers was conducted. Ethics approval for the study protocol was obtained from NRES Committee South Central – Southampton B 12/SC/0643.

### Recruitment and sampling

In this paper we use the term caregiver, defined as ‘anyone who cares, unpaid, for a friend or family member who due to illness, disability, a mental health problem or an addiction cannot cope without their support.’^12^ We identified people with at least six months’ experience of being caregivers, as the research team considered that they would be best able to reflect on what they had learnt formally and informally, and identify any gaps in current service provision. Caregivers were contacted by community-based cardiac nurses or the cardiac rehabilitation team in three geographical settings reflecting the diversity of the United Kingdom population, i.e. Cornwall (a rural, stable older white population) and Birmingham and Leicester (ethnically diverse populations). The clinical team checked that the person with HF and their caregiver were both still alive prior to contact and sent the caregiver a participant information sheet, a questionnaire and a letter inviting them to participate in the research. Caregivers from a support group for people with an implantable cardiac device were also invited to participate by a member of the research team (JW) at one site. Potential participants were also approached through advertising by the National Cardiomyopathy Association.

A questionnaire, collecting demographic and socioeconomic information with a free text section to allow caregivers to provide their contact details, was sent to all participants together with a stamped addressed envelope. A purposive sample of caregivers was selected by the research team (JW, DT) using maximal variation techniques to develop the sampling frame.^18^ The sample represented a mix of demographic and social factors including time as a caregiver, gender, age, socioeconomic status and ethnic diversity.

### Interviews and focus group

Participants were invited to be interviewed at a convenient location in relation to their caregiving responsibilities. Following informed consent, semi-structured interviews were conducted by either JW or DT. An established REACH-HF patient and public involvement group informed the development of the topic guide (Table 1). The researchers used probing, mirroring and non-verbal communication techniques.^19^ The researcher summarised the content of the interview at the end of the discussion and invited the caregiver to add anything else they would like to share about their experience of providing care.

**Table 1.** Topic guide.

Topic Guide for Individual Interviews
How the caregiver reacted to HF and adjusted to the role; how the role is negotiated with the person being cared for?
Formal support received from health and social care services including information about being a caregiver.
How the caregiver looks after his or her self?
Support from family and friends, support groups and forums.
What should be included in the family and friends resource?
What would the caregiver recommend to a new caregiver?
How should the REACH-HF facilitator should work with the caregiver?

The researcher wrote field notes to inform the analysis at the end of the interview, detailing the home environment, geographical location and how the interview was performed; reflecting on their own performance and influence on the interview; how the caregiver responded to the questions and initial thoughts about the main points arising from the interview.

Following completion of the individual interviews, a focus group was conducted with a different group of caregivers in Cornwall alone. The caregivers were identified by the process described earlier. The purpose of the focus group was to confirm the findings from the individual interviews, ensure all significant caregiver needs had been identified, refine the potential content of the family and friends resource and determine the order of the ‘needs’ to be presented in the resource. The researcher (JW) set out the purpose of the focus group and gained informed consent prior to beginning the audio-recorded discussion. JW presented a summary of the interview findings and facilitated the focus group, ensuring that all caregivers had an opportunity to voice their opinion if they so wished. Once the topics for the resource had been introduced, the group was given a set of topic headings based on the thematic analysis and asked to rank them in order of priority for the caregiver resource. DT made notes of the group members’ interactions. Each researcher subsequently wrote their own field notes detailing a summary of the proceeding and initial observations.

### Analysis

The audio-recordings from the interviews and focus group were transcribed verbatim by an experienced transcriber, checked for accuracy by DT and JW, and anonymised. Data were managed using computer software Nvivo 10 and thematically analysed.^17,20^ The researchers conducted a six-step process which involved familiarisation oneself with the data, generating initial codes, searching for themes, reviewing the themes, defining and naming the themes, and producing the report.^16^

#### Individual interviews

Two researchers (JW, DT) independently listened to the recordings of the individual interviews several times and read the field notes to familiarise themselves with the relevant data. Small sections of data were assigned a code or tag that summarised the content either descriptively or interpretively. Codes with common features were grouped together in emerging themes, before finally being assigned to interpretive overarching themes.^16^ Reflexive research memos were used as an audit trail of the analysis procedure.^21^ A third qualitative researcher (JF) conducted independent analysis of each transcript before all three researchers met to discuss and agree the findings. A copy of the transcript was offered to the caregiver participants for comments.

#### Focus group

The focus group data were independently analysed by the construction of simple descriptive summaries by JW and DT. The researcher looked for consensus among the group and any differences were explored by seeking an explanation for agreement or disagreement.

## Results

Twenty-two participants took part in individual interviews, 21 were in the caregiver’s home and one was in the research unit at a general hospital. Interviews lasted between 42 and 87 min with a mean of 62 min. The participants were aged 39–84 years, 16 were women and 20 were in spousal or partner relationships. Sixteen interviews took place in Cornwall, four in Birmingham and two in Leicester (see Table 2). At the request of the caregiver the person with HF was present at, and participated in, 12 of the interviews. The researchers focussed the interviews on the caregiver experiences.

**Table 2.** Demographics of caregiver participants in the individual interviews.

Socio-demographic factor	Participants (N = 22)
Males	6
Mean age (range)	67 years (39–84 years)
Ethnicity	
• British White	18
• Black Caribbean	1
• British Black	1
• Indian	2
Relationship to the cared for person	
• Spouse/partner	20
• Father/son	1
• Daughter/mother	1
Length of time as a caregiver	6 months to 8 years
Employment status	
• Employed	3
• Working age full time caregiver	1
• Retired	18
Caregiver health	
• Fit and active	10
• Some illness or disability (bone and joint, lymphoma, stress)	12
Household annual income	
• Below £10K	4
• £10–20K	11
• £21–30K	5
• £31–40K (one withheld information)	1
Education	
• No qualifications	6
• Secondary education	5
• National vocational qualification or work-related qualification	5
• Degree or higher	6
Home ownership	
• Own home	19
• Social housing	2
• Private rent	1
Area of residence	
• Small city	2
• Village	8
• Town	7
• Suburb of city	5

The focus group consisted of four caregivers and lasted for 130 min. Three were women; all were spouses/partners and aged 42–72 years (see Table 3). During the focus group, one caregiver became unwell and went home. Three caregivers were accompanied by the person they cared for. These people sat together in a separate social group during the focus group but were not included in the research.

**Table 3.** Demographics of caregivers in the focus group.

Socio-demographic factor	Participants (N = 4)
Males	1
Mean age (range)	62 years (42–72 years)
Ethnicity	
• British White	4
Relationship to the cared for person	
• Spouse/partner	4
Length of time as a caregiver	6 months to 6 years
Employment status	
• Employed	1
Caregiver Health	
• Fit and active	1
• Some illness or disability (bone and joint, stress, gastrointestinal)	3
Household annual income	
• Below £10K	2
• £10–20K	2
Education	
• No qualifications Secondary education	1
• National vocational qualification or work-related qualification	2
Home ownership	
• Own home	2
• Social housing	2
Area of residence	
• Village	1
• Town	3

## Caregiver needs

Three distinct overarching themes related to the caregiver needs were identified: providing support, transition to becoming a caregiver and engaging help (Table 4). They encompass needs from formal health and social services and informal support from family, friends and voluntary agencies.

**Table 4.** Caregiver needs.

**Caregiver Needs for the Family and Friends Resource**
**1. *Providing Support***
**Understanding heart failure**
Caregiver understands condition (e.g. what it is, causes, which type, expectations/prognosis) and how treatments work
**What to do if there are signs and symptoms of HF**
Supporting the patient to monitor and manage signs and symptoms, but stepping in/taking over when appropriate (and as agreed with the cared for person)
a) Good days – knowledge and understanding about signs and symptoms, knowing what is normal (developing realistic expectations for self and for cared for person, having ‘good days and bad days’). Ability to take appropriate action (e.g. managing fatigue, managing breathlessness).
b) Bad days– danger signs, extra monitoring and ability to take appropriate action (e.g. seeking help, changing diuretic dose under the direction of the health care professional)
**Dealing with emergencies**
What can the caregiver do in an emergency, e.g. calling emergency services, resuscitation techniques, when to resuscitate, when not to resuscitate
**Understanding and managing medicines**
Routine for medication ordering, pill box, prompting and recording
**Providing emotional support**
Caregiver provides emotional support for the cared for person, acknowledging and managing mood swings
**Promoting physical activity**
What types of physical activity, how to assess when the cared for person does too little or much, etc. Understanding what is a therapeutic level of physical activity to develop or maintain fitness. Focus on caregiver resource will be about how the caregiver can facilitate the process or maintain it.
**Providing personal care**
Practical tips (e.g. moisturise skin, help with hygiene, dressing)
**Living with a cardiac device**
Understanding cardiac devices and building confidence to get out and about
**Helping someone feeling low or depressed**
Knowing where and when to get help
**2. *Becoming a caregiver***
**Becoming a caregiver**
Psychological aspects of becoming a caregiver, negotiating your role (support without smothering, etc.), transition to becoming a caregiver, role reversal, discussions with the cared for person (developing congruence), etc. Managing change in self-concept. Trips out, family activities and events, being part of a group, volunteer activities, employment, being a grandparent
**Communication with health professionals**
Caregiver attends consultations, is involved in discussions, facilitation, communication training, signpost to community matrons, heart failure nurses
**Managing caregiver mental well-being**
Identifying when stress is problematic, engaging social support, managing caregiver’s low mood, anxiety, identification of depression and signpost to appropriate care providers, etc.
**Managing own physical health**Including physical activity, monitoring own health and seeking help/advice when appropriate. Preventative measures such as safe lifting, dietary information and practical meals advice (easy and quick and healthy)
**Sleep**
Assessing and dealing with caregiver sleep issues
**Manage /organise the home and work environments – daily hassles**
Calendar / diary, prioritisation of tasks, time management, getting help, flexible work hours
**3. *Getting Help***
**Social support – engagement**
For example, friends, relatives, carer groups, etc.
Information /planning support, emotional support, practical support, respite care, age concern sitting service, community groups, Age UK, caregiver organisations
**Social services – engagement**
Home needs assessment, getting a carer assessment (stress, access to carer support services)
**Manage financial burden/organising benefits**How to obtain benefits, where to go for help with forms
**Living with uncertainty**
Variable symptoms, possible deterioration/de-compensation events, end-of-life issues, spiritual care

**Figure 1.**
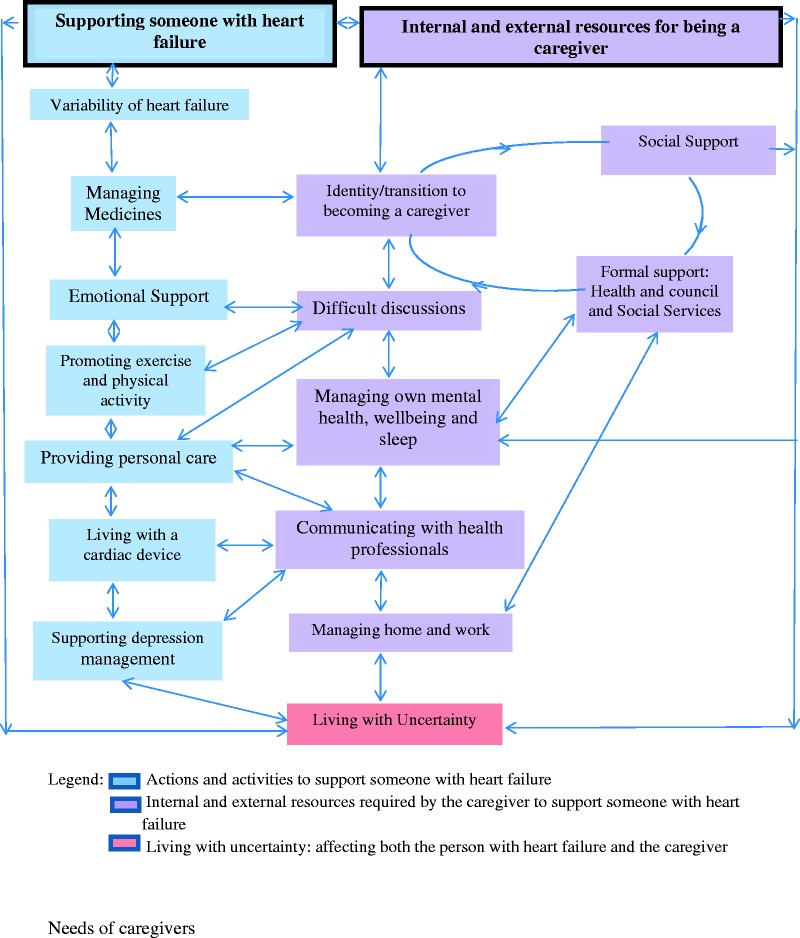
Conceptual framework of caregiver needs in heart failure management.

From the analysis of the discrete themes and the relationships between them, a conceptual framework (Figure 1) was developed that illustrates the routes by which health professionals can target support to meet the needs of caregivers as they ultimately live with the long-term uncertainty of HF.

### Providing support

The first priority for caregivers was how to care for someone with HF, especially at the time of diagnosis. There was also evidence of ongoing uncertainty about HF management, particularly for those who had not seen a specialist HF nurse or attended cardiac rehabilitation.

#### Variability of HF

A specific challenge was in coping with the variability of HF symptoms and many reported there was no average day as a caregiver. This had a direct impact on their role as it required them to discern when to encourage self-management and physical activity or when to step in and take control. There was evidence of ‘hyper vigilance’, constantly monitoring (day and night) for signs and symptoms of HF even when the person they were caring for is well.

‘Definitely you watch, definitely, even maybe from a different room you watch’ (P15).

Knowledge of how to support someone with HF came from a variety of sources and was learnt experientially including by watching community HF nurses. Learning skills was a complex process as it is dependent on the reaction of the person with HF and their willingness to take action and manage the condition.

‘It’s just experimentation at the minute, to see how far he can go… you haven’t got a, slide rule saying, well he should be here so far and so on [ ]. I suppose it also depends on the person themselves; their willpower to get on, or their won’t power’ (P12)

Many caregivers had seen the person they were caring for in an emergency situation and reported that they required information about what to do in an emergency. This included how to recognise when signs and symptoms need urgent attention and how to perform cardiopulmonary resuscitation.

#### Understanding and managing medicines

There was much concern about medications especially when the person they were caring for was first diagnosed, discharged from hospital, became unwell or if medication was changed, as frequently occurs in HF management. Medication was frequently complex and as a result most caregivers developed a structured system to ensure there was an adequate supply of medicines. One caregiver was managing 35 tablets a day as the person being cared for also had other co-morbidities. The most frequent activity identified by the caregivers was to remind the person with HF to take medicines, as they realised that the person they were caring for had memory problems. This could be done by telephone or text messaging if required.

Learning about medication happened over time and knowledge came from a variety of sources including consultant cardiologists, specialist nurses, general practitioners, drug information leaflets and the internet. One woman had an exceptional knowledge of the medicines and used information from the internet to inform her discussions with the consultant.

Information about record keeping was identified as a need by many caregivers as the person with HF may see multiple health care professionals and have difficulty recalling events or when and why changes were made to medicines. A minority of caregivers had detailed diaries of events, lists of effects and side effects, when and why, and by whom medicines were changed.

‘I write out a checklist, so I know what tablets I’ve got to put in (pill box)… if put like a chart in your manual as to what tablets have to be taken when’. (P19)

#### Providing emotional support

Many caregivers reported that they provide emotional support and encouragement at the same time as managing their own emotions.

I try and keep her morale high by saying ‘If we take things calmly then things are alright.’ But obviously she knows and she’s accepted the situation, she’s in a terminal situation.…..It was hard at the beginning, a lot of tears and crying and that sort of thing. (P8)

#### Promoting exercise and physical activity

There was a positive attitude towards physical activity although many reported that they needed more specific information about what type and level of activity is required. A minority of caregivers who had not received specific advice about exercise had developed their own programme involving walking with the person with HF.

#### Providing personal care

Most people with HF were relatively independent at the time of the interview, although some were physically dependent on the caregiver, and others had been in the past. We noted that caregivers who were providing physical care were concerned about skin integrity and manual handling and had learnt how to manage these by watching nurses who came to the home or from lay sources of knowledge.

#### Living with a cardiac device

Nine caregivers reported that the person they were caring for had a cardiac device. There was concern for those who had a defibrillator about what to do if the device fired even when information had been given.

‘I think I put my head in the sand a bit when he first had the ICD….because they were all talking about the shocks. And I thought I don’t know what to do; what shall I do if he has a shock?’ (P15)

#### Supporting depression management.

Depression was a condition some caregivers were supporting. This was seen as distinct from providing emotional support and the focus group members recommended separating this information from providing emotional support.

One weekend, he said, ‘Oh, I can’t stand this any longer. They’ve got to do something else.’ He started talking about finishing it. And then we went to the doctors and he was put on anti-depressants, and it’s helped. (P4)

### Transition to becoming a caregiver

There was a range of reactions to becoming a caregiver from a stoical, accepting belief that it is part of getting older and integral to marriage, to anger and frustration about the loss of personal identity and reluctance to accept a new identity as a caregiver. Some felt alone and burdened.

When they’re having a sleep or when they’re having a bad day, sometimes you just wish you could talk to somebody that understood, that has been there.…It just all gets a bit much sometimes. You do feel very, very alone. (P13)

Caregivers suggest that in adjusting to their role, they need to know that adjustment takes time, with a process of sense making (understanding the personal significance of being a caregiver), experimenting (finding information and learning what works or does not work) and making being a caregiver part of daily life.

#### Difficult discussions

Most caregivers either had health problems of their own or had other responsibilities such as caring for children or employment. Those who appeared to have coped better had discussed the impact of HF with the person they were caring for and clarified what their role should be.

Involve your partner in what you are doing, discuss things; it’s important to communicate, discuss things and then be patient. You’re going to have these highs and lows. (P8)

#### Communicating with health professionals

Caregivers saw many different health professionals including doctors in primary and secondary care, ward and specialist cardiac nurses, physiotherapists, occupational therapists, pharmacists, podiatrists and dieticians. This was, for many, an important activity, especially when the person with HF had difficulty recalling consultations.

I would be lost without, our heart failure nurse, and, all the other input we’ve had from all the other professionals, like the podiatrist and GP…… You can do it, but in partnership with everybody else*.* (P23)

Caregivers want to know about treatment options and contribute to decisions as they believe they know ‘the patient best’. There was evidence of concern, frustration and stress when caregivers had been excluded from consultations either by health professionals or the person with HF. Caregivers stated that they do not always have the same GP as the person they are caring for, which could further obscure the needs of both the cared for and the caregiver.

#### Managing mental health, well-being and sleep

Although stress was not formally assessed as part of the study, there were many examples of stress; not all caregivers had learnt to find ways of managing their mental health and physical well-being.

It can pull you down really, to be honest, and there is no quick answer.… I would definitely just advise [caregivers] to do something, on a physical thing for themself, even if it’s once a week. (P24)

Caregivers often but not always used social support networks for friendship and emotional support including support groups and physical activity such as swimming, running or dancing. Relaxation was often achieved by completing puzzles or reading.

A recurrent problem for caregivers was sleep management. At night they were disturbed by the need to assist with toileting, or were awake worrying about their problems, or checking whether the person was still alive.

#### Managing home and work

Managing daily life was difficult for many, adding a sense of enduring burden. Caregivers who adopted a flexible approach appeared to find it easier to cope with the unpredictability of HF and therefore their day.

You have to learn to go with the flow….It’s no good thinking, right, tomorrow I’m going to do so and so, because nine times out of ten, so and so doesn’t happen. (P4)

### Engaging help

Help came from a variety of sources including from social support (friends and family), the voluntary sector or from formal health and social services.

#### Social support

Engaging help was frequently difficult as caregivers often felt they should be able to cope or were concerned about being a burden to others. Those who were able to ask for help benefited from doing so.

I have always been a bit independent and stubborn…but you need to ask for help, and that is really hard, even if it’s from a close relative or friend…..but once you’ve done it, it’s like well I’m glad. (P4)

Text messaging was frequently used for maintaining social support systems as well as keeping in touch with the cared for person when they were apart. Practical support was also appreciated where there was significant ill health of either the caregiver or the cared for person, commonly this was a sitting service, collecting prescriptions or help with housework.

A few caregivers used voluntary services including faith organisations, the British Heart Foundation or the Cardiomyopathy Association, and the Bureau for Citizens Advice service for information, advice or support but this was not the norm.

#### Formal services

Some help came from formal services including health and social services, although caregivers frequently did not know which services were available or how to ask for help. Few caregivers knew that they have a legal entitlement to a formal ‘Carer’s Assessment’. Many caregivers had a poor experience of coping with the benefits system.

#### Living with uncertainty

Many caregivers were concerned about the long-term future, especially those who had witnessed the person they were caring for become significantly unwell and recognised that death could occur suddenly. They had questions about coping with end-of-life care and how they would cope alone when the person they were caring for had died.

I was frightened.… how I was going to cope if he was taken from me, shall we say? (P2)

## Discussion

### Summary of main findings

Caregivers for people living with HF want to, and are well placed to, support HF management but have many needs to do so effectively. Many caregivers are supporting people with HF and formulating strategies to meet their own needs. Our findings provide a detailed understanding of the caregiver’s needs in HF management (Table 4 and Figure 1). We propose a conceptual framework that maps the many internal and external interlinked resources required in order to support caregivers for people with HF (Figure 1). This illustrates the complexity of caring for a person with HF and the routes by which health professionals, as part of a health and social care team, can target the support that caregivers require. If caregiver needs are met, these results suggest that caregivers can successfully support self-management in HF. When caregivers in this study felt supported, they reported feeling valued and a sense of satisfaction with their role but many did not feel their needs were being met.

### Strengths and limitations

This is the first targeted qualitative study in the United Kingdom that we are aware of, which identifies the specific needs of caregivers for HF management beyond carer burden to inform the development of a specific home-based intervention for caregivers. It includes caregivers from ethnic minorities, those in both rural and urban locations and those who were in employment or with other responsibilities. The sample includes caregivers who were caring for people with HF who were independent, requiring little support as well as those who required physical personal care and assistance with mobility.

A significant challenge was identifying caregivers to participate in this research, as there is no regional or national United Kingdom register of caregivers in HF management and we were therefore, largely reliant on health professionals identifying caregivers. There may be an important subgroup of ‘hard to reach’ caregivers who are not represented in this study (e.g. those with diagnosed depression) who may have additional needs beyond those captured here. Although the study had only one focus group, we felt this was sufficient as, it was able to confirm the findings of the individual interviews. It identified the priority of needs (Table 4), which informed the development of the conceptual framework (Figure 1), and ultimately the family and friends resource. The study may have benefitted from an additional group to include representatives from ethnic minorities; however, the individual interviews from ethnic minority caregivers did not identify specific cultural needs that differed from the white British group.

The person with HF was present during 12 of the interviews and this may have affected the data gathered. We observed as Taylor and de Vocht^22^ indicate that frequently partners can corroborate stories, challenge points raised or introduce new ideas. Caregivers were frequently encouraged by the person with HF to speak freely about the challenges they faced and what they felt about their role. The joint interviews allowed the researcher to gain insights of how couple worked together or in conflict to manage HF and their situation. The presence of the person with HF also gave us valuable context data about the effect of HF on the individual and we witnessed many of the caregiving activities described by the caregivers. We also noted that those who wanted to be interviewed alone found a way to do so.

### Comparison with existing literature

HF patients often delay seeking help^23^ and may be reliant on caregivers to identify when their condition deteriorates.^16,24^ This study shows that caregivers need to know how to monitor the person they are caring for and when to take action if required which may reduce the delay in seeking help.^25^ Part of the role therefore, includes communication with health professional, yet as we found, caregivers often report feeling they were not involved in consultations.^26^ Involving caregivers in consultations is important as caregivers can also increase the compliance of people with HF to taking medicines.^9,13^ This study also identifies specific needs related to information and practical advice on managing medicines.

How caregivers support a person with HF is affected by the responses of the person with HF to the condition. This reflects the findings of our qualitative synthesis.^16^ Previous research with couples concluded that those who seemed to cope most effectively were those who had discussed HF, its management and the effect it has on both parties.^27^ Our research also included parents and adult children, as both cared for and caregivers, and reached the same conclusion. Management of chronic disease occurs in the context of a wider community of formal and informal networks as self-management or self-care is a joint activity between the individual, their families and the community.^28^ Caregivers have been found to have a lower quality of life than in the general population and as the person with HF deteriorates, adjustment to being a caregiver is therefore, a continual process. As we have demonstrated, caregivers require an extensive network of formal and informal support.^28^^–31^

Piamjariyakul et al.^32^ conducted eight focus groups with HF patients and health care professionals and 17 telephone interviews with caregivers to inform the development of instructional materials for a telephone-based coaching programme on managing HF. They identified seven main themes relating to HF home management; caregiver involvement: continuous learning about HF, acceptance of and coping with the HF diagnosis, learning from other patients, guidance for practicing self-management and for daily problem solving, lifestyle change and financial resources. In addition, our study has identified the complexity of caring for a person with HF and the dynamic and interacting nature of the needs of caregivers. In order for the caregivers to provide support, they must also be supported to address their own physical and mental health needs and mobilise an informal and formal support system.

## Implications for clinical practice and research

Caregivers should be actively included in treatment and care planning decisions, as this can improve HF self-care, but also because it can facilitate and improve communication with the health care professional.^33^^–35^ Involvement in care decisions could mediate the negative effects of caregiving.^26^ As recommended by Clark et al. we suggest that consultations and HF interventions should include understanding about HF, discussions about goals, treatment options, address patient and caregiver expectations, desires and capabilities, social and formal support options.^5^ This is particularly important as the whole family prepares for end of life. There is evidence that although health care professionals are often uncomfortable with end-of-life discussions patients want an opportunity to prepare for end of life. Discussions could include fears and directives about resuscitation and deactivation of devices.^34,35^ Caregivers should be involved in the discussions as decisions also affect their role and we suggest that these discussions could be best placed in primary care with opportunities to discuss fears and advanced care planning.

This study demonstrates that many caregivers are willing and able to support self-management strategies once they are equipped with the required knowledge and skills which comes through appropriate communication with health professionals, experiential learning and lay sources of support. Policies and clinical practice should ensure that caregivers are equipped to support the management of HF and meet their own psychological and physical needs as they adjust to becoming caregivers.^5^ This may be a challenge as caregivers may hide their emotions and responses, as they feel they have a duty to care. Caregivers require personal, social and formal resources to meet their needs and to care for a person with HF. Despite this, many caregivers in the UK are unaware of their legal entitlement to a ‘Carer’s Assessment’. Health professionals should therefore ask each patient with HF if there is someone who is supporting them and make a referral for a ‘Carer’s Assessment’ where this is required or refer to other agencies such as mental health services. Primary care health professionals are well placed to ask patients if they have a caregiver or have caregiving responsibilities as part of planning care and to have a register of caregivers. This may be a challenge as some caregivers believe their activities are part of being in a relationship and do not identify themselves as caregivers. Community-based health professionals including HF specialist nurses and pharmacists could offer information and advice. Caregivers should be included in discharge planning from hospital, especially where there have been significant changes to medication.

This research is part of a National Institute of Health Research funded REACH-HF programme to develop and evaluate a home-based facilitated rehabilitation manual aimed at people with HF and also their caregivers.^7^ The results of this study have therefore informed the development of a Family and Friends Resource which is being formally assessed through a multi-centre randomised controlled trial that started recruitment in January 2015. There is scope for further research as the resource may be adapted for caregivers with specific needs including caring for someone with HF who is approaching end-of-life care and caregivers with specific mental health problems such as depression. We believe this qualitative study of the needs of caregivers has important implications for other long-term conditions for which further research is required.

## Roles

HD is a REACH-HF Co-Programme Investigator contributing to the design and management of the study, JW, KJ and NB designed the study, JW and DT collected the data and undertook the analysis. JF undertook analysis. JW, NB and JF wrote the manuscript. CG, KJ and CA contributed to the conception of the study. All authors made comments on the participant materials and the manuscript.

REACH-HF (Rehabilitation Enablement in Chronic Heart Failure) Rod Taylor (Programme Co Lead), University of Exeter, Hayes Dalal (Programme Co-Lead), Charles Abraham, Jackie Austin, Nicky Britten, Russell Davis, Patrick Doherty, Lorna Geach, Colin Greaves, Heart Manual Office Edinburgh, Kate Jolly, Kevin Paul, Peninsula Clinical Trials Unit, Sally Singh, Robin van Lingen, Jennifer Wingham.
